# The Impact of Manufacturing Imperfections on the Performance of Metalenses and a Manufacturing-Tolerant Design Method

**DOI:** 10.3390/mi13091531

**Published:** 2022-09-16

**Authors:** Yicheng Zhu, Wenjuan Wang, Feilong Yu, Qingquan Liu, Zilu Guo, Guanhai Li, Pingping Chen, Wei Lu

**Affiliations:** 1 State Key Laboratory of Infrared Physics, Shanghai Institute of Technical Physics, Chinese Academy of Sciences, 500 Yu-Tian Road, Shanghai 200083, China; 2 University of Chinese Academy of Sciences, No. 19A Yuquan Road, Beijing 100049, China; 3 Shanghai Research Center for Quantum Sciences, Shanghai 201315, China; 4 School of Physical Science and Technology, ShanghaiTech University, Shanghai 201210, China

**Keywords:** optoelectronic integrated devices, metalens, dry etching

## Abstract

Metalenses play an important role in optoelectronic integrated devices, given their advantages in miniaturization and integration. Due to its high aspect ratio subwavelength structure, fabricating metalenses requires a high-level dry etching technology. Consequently, structure deformation of the metalens will exist if the etching process of the material is not mature enough, which will impair the metalens’ performance. In this paper, a polarization-independent InP dielectric metalens is designed to focus the incident light from air into the substrate, which is used for monolithically integrating with the InGaAs/InP photodetector in the future. Subsequently, with the simulation method, we investigated the impact of the structure deformation on the metalens’ performance, which was found in our InP dry etching process development. We have found that the sidewall slope and aspect ratio-dependent etching effect greatly impaired the focusing efficiency because of the phase modulation deviation. To solve this problem, we proposed a manufacturing-tolerant design method, which effectively improved the performance of the device with structural deformation. Our work is instructive for developing metalenses and can accelerate their integration application.

## 1. Introduction

In the past decades, metasurfaces that can precisely and freely regulate various properties of the light field have been extensively studied [1,2,3,4,5,6]. Compared with traditional optical components and three-dimensional (3D) metamaterials, metasurfaces have advantages in miniaturization and integration. These make metasurfaces bring a significant revolution to the photonic and optoelectronic integrated devices in pursuit of miniaturization, multifunctionality and high coupling efficiency [7,8,9,10]. For optoelectronic integrated devices, the metalens as a kind of metasurface can be considered the most promising and basic component [11,12].

To obtain high signal-to-noise ratio optoelectronic devices, lenses are often used to integrate with the photodetectors to converge the incident light into the reduced active region of the photodetectors. As for the lenses integrated with detectors, the focal length, the focal spot size and the focusing efficiency are particularly important. It is necessary to concentrate the incident energy into the few-microns-thick absorption layer precisely. At the same time, the size of the active region needs to be as small as possible, which is limited by the size of the focused spot diameter to a certain extent and the fabrication process.

The current commercial integration method is to attach the detector and the spherical microlenses together [13,14,15]. However, on the one hand, the fabrication of the spherical microlens is complicated and the controllability of the spherical surface profile is quite difficult [16,17]. On the other hand, the alignment of this method is complex and may introduce an excess loss of efficiency [18,19,20]. To solve these problems, it has been proposed to fabricate all-dielectric metalenses directly on the detector’s substrate to achieve monolithic integration, which was named solid-immersion metalenses for focusing light from air into the substrate material in reference [21]. Fabricating metalenses requires a high-level dry etching technology due to their high aspect ratio subwavelength structure. At present, the mainstream materials are silicon (in infrared wavelength) and TiO_2_ (in visible wavelength) due to their relatively advanced processing technology [22,23]. Nevertheless, some bottlenecks will exist when integrating them onto detectors, especially infrared photodetectors. Since the 2π phase coverage is required for freely controlling the wavefront, a sufficient thickness medium is needed. According to the report, a metalens working in the long-wave infrared wavelength demands at least an 8-μm-thick silicon [24]. However, deposing such thick amorphous silicon with good uniformity and adhesion on the substrate of the detector is quite difficult, and it is more difficult to process them further [25,26]. According to the above, the use of detectors substrate materials with a more immature etching process has become a compromise solution for metalenses’ monolithic integration. With an immature etching process, a deviation between the actual and the designed structure cannot be avoided, such as sidewall slope on pillars, rough surfaces in trenches and the aspect ratio dependent etching (ARDE) effect, which causes narrow features to be etched shallower than large features [27,28,29,30]. These structure deformations will affect the performance of the metalens more or less, which has been seldomly studied before.

In this paper, we mainly focus on the impact of structure deformation introduced by the etching process on the focal length, the size of the focal spot and the focusing efficiency. We first designed a polarization-insensitive metalens with InP material for monolithic integration, whereas InP is the substrate material of InP/InGaAs near-infrared photodetectors with an extremely low absorption rate and high refractive index in near-infrared [31,32]. Since the etching process of InP material is not mature enough to meet the design requirements, we studied the effects of structure deformation mentioned above through the 3D finite difference time domain (FDTD) method in combination with the actual process conditions. In the end, we put forward a manufacturing-tolerant design (MTD) method to correct its phase control deviation and verified this method through the FDTD simulation. The MTD method is of great significance for reducing the cost of process development and improving the device’s performance. Our work is instructive for developing metalenses and can accelerate their integration application.

## 2. Results and Discussion

### 2.1. Design and Characterization of the Solid-Immersion Metalens 

**Figure 1:**
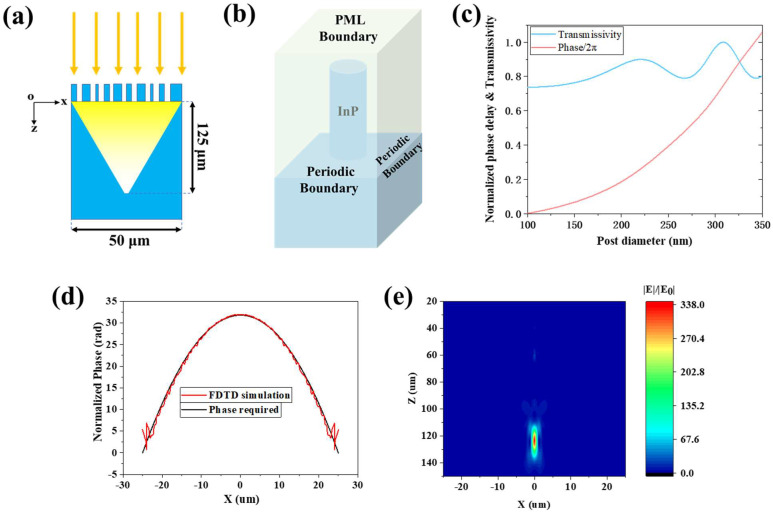
(**a**) Schematics of the InP solid-immersion metalens sectional view. (**b**) The setup of the unit cell simulations. (**c**) Simulated transmissivity and phase of the unit structure as a function of the post diameter with period of 400 nm. (**d**) The required phase profile (black curve) and the computed phase profile of our design (red curve) along the x-axis cutting through the center of the lens. (**e**) Simulated relative E-field intensity profiles of the metalens in the xz-plane (x from −25 μm to 25 μm, z from 20 μm to 150 μm).

To integrate onto the InP/InGaAs near-infrared detectors, we designed a solid-immersion metalens working at incident wavelength λ = 1550 nm with InP material. The refractive index of InP material is about 3.17 and the absorption is negligible. As shown in Figure 1a, the light focuses into the InP substrate after passing through the InP metalens on the top, of which the focal length is 125 μm. The metalens occupies a 50 μm × 50 μm square, which consists of nanopillars of the same height. The design of the metalens consists of three steps, which is similar to the method mentioned in reference [33]. In the first step, we calculated the phase profile of the lens. In order to meet our application requirements, the phase profile of the metalens needs to follow the equation:

(1) $$\varphi \left(x,y,\lambda \right)=C-\frac{2\pi }{\lambda }\cdot n\cdot \left(\sqrt{x^{2}+y^{2}+f^{2}}-f\right)$$

where *λ* is the wavelength, *C* is a constant, *n* is refractive index of the substrate and *f* is the focal length. Metalens based on our design is a kind of binary subwavelength gratings. When the period of the gratings is small enough, the high-order diffracted waves become evanescent waves, and only the 0-order diffracted waves are transmitted and reflected. The phase of the transmitted zeroth-order light is determined by the fill factor of the grating as well as by the period/wavelength ratio and the grating depth. Here, the required phase profile is achieved by varying the diameter of the dielectric nanopillar units, in which the waveguiding effect is the dominant mechanism [33]. In the appropriate condition, each nanopillar unit can be approximately considered to modulate the local phase over its occupied subwavelength area [34], and the relation between the unit and modulation phase is given by:

(2) $$\varphi =\frac{2\pi h}{\lambda }n_{eff}$$

where *h* is the height of the nanopillar and $n_{eff}$ represents the equivalent refractive index of the unit cell composed of the nanopillar and its surrounding medium. The $n_{eff}$ varies as the diameter of the nanopillar changes, and then the value of φ changes. In the second step, in order to find the relation between the phase and diameter, the performance of the nanopillar unit cells were characterized by the FDTD method (Ansys Lumerical FDTD). As shown in Figure 1b, periodic boundary conditions are employed in the x and y direction while PML boundary condition is set in the z-direction. We set the period at 400 nm, InP pillar height at 1.5 μm and scanned the pillar’s diameter from 100 to 350 nm. The results are shown in Figure 1c. Considering manufacturing constraints, we chose pillars with diameters ranging from 100 to 350 nm to fulfill the phase coverage requirements. In the third step, we discretized the area into unit cells (xi, yi) with period of 400 nm. At each position (xi, yi), a pillar with an appropriate diameter is chosen to impart the required phase mask φ(xi,yi), which is shown in Figure S1 (Supplementary Materials) [35,36]. According to the steps above, the design of the solid-immersion metalens was completed and the full device simulation was carried out in Lumerical FDTD Solutions. Figure 1d shows the required (black curve) and the computed phase profile (red curve) along the x-axis cutting through the center of our designed metalens. The red curve oscillates near the black curve while the oscillation is greater at the edges than at the center. It is because the required phase at the edge varies more rapidly than at the center, and a smaller spacing of the units is required to make the phase finely sampled, which is not considered in our design [37]. Moreover, the existence of high-order diffraction at the edge is hard to control. Figure 1e is the relative E-field intensity (|E|/|E_0_|) distribution in the xz-plane, where |E_0_| is the Electric field amplitude of the source power. The focal length of the metalens is about 123.5 μm, close to the designed value (f = 125 μm). The full width at half maxima (FWHM) of the focal spot is 1.1 μm or 0.71λ. Its transmissivity is about 85.8%, and the focusing efficiency is 62.3% (NA = 0.705), where the definition of focusing efficiency is the same as that mentioned in reference [38]. Because of the material’s low absorption, the transmittance loss mainly comes from reflection. Moreover, the focusing efficiency is close to that of the CdZnTe metalens (focusing efficiency = 63%, NA = 0.962) mentioned in reference [38].

### 2.2. Impact of the Manufacturing Imperfection

**Figure 2:**
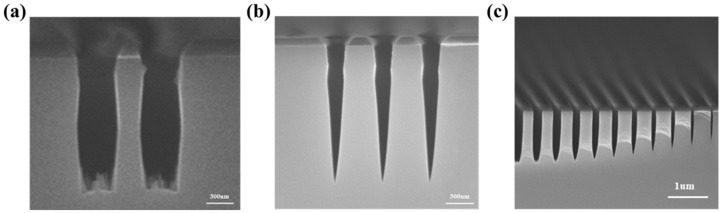
The common phenomena in our etching process development: (**a**) Rough surface in grooves. (**b**) Sidewall slope (~85°) on pillars. (**c**) The ARDE effect in dry etching.

Compared to the designed structures in Section 2.1, the actual structure is much more complex, mostly caused by the etching process in manufacturing. It will affect the property of the whole device. In this section, we studied the InP metalens performance degradation brought by the imperfect etching process in some common situations, such as the rough surface in trenches (Figure 2a and Figure S2), the sidewall slope on pillars (Figure 2b) and the ARDE effect (Figure 2c). Figure 2c is the section image of the etched pillars and Figure S3 is the top view image. They all existed in our etching processes of InP material. In this section, we studied the effects of structure deformation mentioned above through the FDTD method.

#### 2.2.1. Impact of the Rough Surface in Trenches

As for the dry etching process of InP material, except for some extreme cases [39], the RMS surface roughness can be controlled under 5 nm in most cases. The low-performed process can also control the roughness under 30 nm, while the well-performed under 0.2 nm [40,41]. Taking these actual situations into consideration, we modified the models built in the simulations in Section 2.1 by adding a rough surface on the substrate with RMS roughness of 5 nm and 30 nm in two cases. The rough surface module comes from the Lumerical FDTD solutions software. It is generated by creating a random matrix of values in K space. A Gaussian filter is applied to this matrix, and then a Fourier transform is used to transform the matrix back to real space.

**Figure 3:**
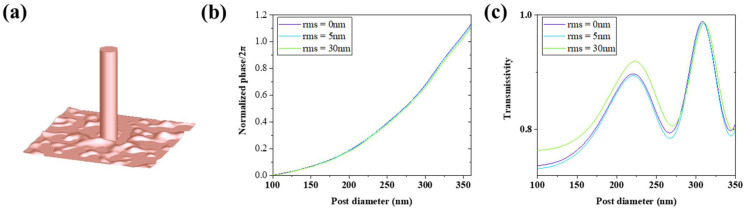
(**a**) One of the models of unit structures with rough surface (rms = 30 nm) in trenches established in software. (**b**,**c**) Simulation results of units with different surface roughness in trenches.

Then we simulated the units (Figure 3a) and the solid-immersion metalens with the rough surface in trenches as Section 2.1 did. As shown in Figure 3b,c, the rough surface has little impact on the phase shift and transmittance of the nanopillar units. Moreover, the rough surface improves the unit transmissivity in some cases, surprisingly. This may be caused by the periodic boundary conditions in simulation so that the rough surface is also considered a periodic structure with the same subwavelength period. Then, we simulated the metalens with a rough surface, and we found that their transmissivity was not affected by the roughness much where the transmissivity was 85.8% (RMS = 5 nm) and 85.3% (RMS = 30 nm). Little light is scattered by the rough surface in these conditions. Their focusing efficiency is 61.3% (RMS = 5 nm) and 60.5% (RMS = 30 nm). Respectively, they are 1.7% and 2.9%, slightly lower than the ideal structure. Moreover, their focal length remains unchanged. According to the above, we considered that the rough surface in trenches (rms < 30 nm) caused by the common dry etching condition does not have much impact on the performance of metalenses. The reason may be that the rough surface undulations have a small proportion of the whole structure undulations where the posts are 1.5 μm high.

#### 2.2.2. Impact of the Sidewall Slope and Aspect Ratio Dependent Etching (ARDE) Effect

Metalenses consist of high aspect ratio unit structures. As for such precise structures, inclined sidewalls and the ARDE effect will appear more or less. Since the actual fabricated structure is more complicated, we try to simplify the structure model at first.

**Figure 4:**
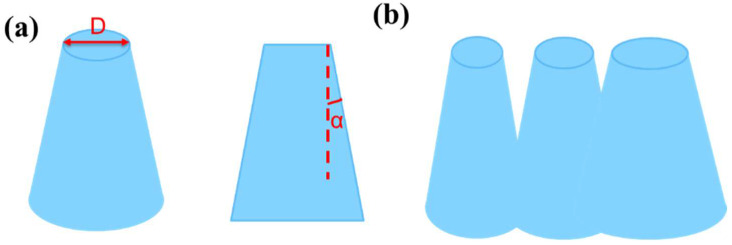
(**a**) Schematic of the model built with the truncated cone to simulate sidewall inclination. (**b**) Adjacent unit structures will overlap when the diameter of the bottom exceeds the unit period length.

We used the truncated cone to approach the actual structure with irregular inclined sidewalls. As shown in Figure 4a, we defined the top diameter of the truncated cone as D and the angle between the sidewall and the vertical direction as α. Generally speaking, the top shape of the structure is the most similar to the mask among other parts of the structure. What is more, its controllability is also the highest, which can be easily adjusted by modifying the layout or exposure parameters. In comparison, the shape of the other parts is more impressionable by the etching process, which may cause them to deviate considerably from the design. Therefore, we constructed the unit structures by setting the diameter D and angle α in simulation, which largely imitates the process from designing the layout to fabricating the actual structure. It is worth mentioning that as D and α increase, other parts of the truncated cone, especially the part near the bottom, may exceed the period range, even if the parameter D is controlled within the period. As Figure S4 shows, the yellow region indicates that the base of the cones exceeds the unit period. This makes it possible to connect parts of adjacent units when building the whole device structure, as shown in Figure 4b. What is more, the larger value of D and α will result in a shallower trench area without InP material. This feature is quite similar to the ARDE effect. As the ratio of the depth to the width of the feature increases, it becomes increasingly difficult for the reactants to diffuse the bottom of the feature as well as reaction byproducts to diffuse out and be pumped out of the chamber. It behaves as narrow features etched shallower than large features. Therefore, with the fixed periodic structure, the bigger diameter of the pillar will cause the smaller trench width, and the etch depth will decrease. According to the above, our established model includes both sidewall slope and ARDE effect phenomena. Moreover, this model is quite similar to the structure morphology we fabricated in our experiment shown in Figure S5.

**Figure 5:**
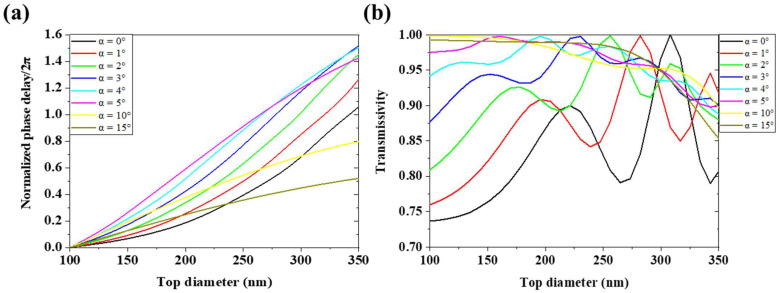
Simulation results of units with different values of α. (**a**) Relationship between D and phase shift. (**b**) Relationship between D and transmissivity.

Taking our fabrication experiment into consideration, we simulated the unit structure with α from 1° to 5° for common fabrication processes and α of 10° and 15° for studying the samples with larger deformation as special cases. The units’ height and period are the same as those in Section 2.1. As shown in Figure 5a, the sidewall slope produces a significant deviation in the units’ phase modulation performance even in the condition α = 1°, where the deviation is much greater than that caused by the inferior rough surface (rms = 30 nm). The units’ phase coverage first increases and then decreases with increasing α. Moreover, when α is equal or greater than 2°, some of the unit structures exceed out of the period region as Figure S4 shows. In this condition, a truncated structure is taken into simulation and the units’ phase modulation was affected significantly by sidewall slope and the ARDE effect at the same time. What is more, the phase coverage of the units is greatly reduced at α = 10° and α = 15°, where the units can no longer provide 2π phase coverage. This results from the units’ filling factor varying in a smaller range when α is equal to or greater than 7.6 degree. In this condition, all the units will merge with their neighbors, whatever D is, and the trench depths of all the units do not meet the design requirements. It proves that a trench-depth-limit etching process will reduce the phase coverage and cannot fabricate a device that can freely control the phase of the wavefront as designed. Figure 5b shows that as α increases, the transmittance of most units increases slightly. This implies that within the condition of our choice, the device composed of units with more sloping sidewalls may have higher transmissivity. Although the unit-cell structures in the simulation above are not completely the same as those in the whole device simulation when the adjacent units connect together, these structures’ model can be regarded as an approximation.

**Figure 6:**
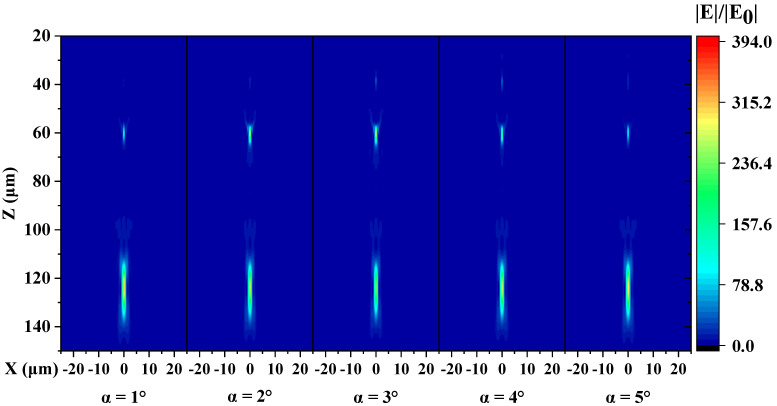
Simulated relative E-field intensity profiles in the xz-plane of metalenses composed of units with different values of α.

Then, metalenses composed of units with α of 1°–5° were simulated. Figure 6 shows the simulated E-field relative intensity distribution of the metalenses with different values of α. It should be noted that we only simulated the metalens composed of the truncated cone with α of 1°–5° because 2π phase coverage cannot be provided by the unit when α is 10° and 15°. From the results, the focal length does not vary much as the sidewall slope varies from 1° to 5° and remains around 123 μm (designed 125 μm). Moreover, the FWHM of the focal spots remains 1.1 μm. But the intensity of the primary focal spot (PFS) declines, and a secondary spot, even a tertiary spot exists.

What exceeds our expectation is that the intensity of the primary focal spot and the focusing efficiency does not decrease monotonically with increasing angle. When α changes from 1° to 3°, the PFS intensity gradually decreases while the secondary spot intensity increases. When α changes from 3° to 5°, the trend reverses instead. Moreover, the trend of focusing efficiency is the same. When the sidewall deviation angle α is 1°, the focusing efficiency of the lens is 61.4%, which is only 1.4% lower than that of the ideal structure. As α increases to 2°, the efficiency significantly reduces to 50%, decreasing by 19.8% compared to the ideal structure. As α increases to 3°, the efficiency reduces to 44.3%, the lowest in our simulations. As α increases from 3° to 5°, the efficiency grows to 54.5%, which is still much smaller than the ideal one.

**Figure 7:**
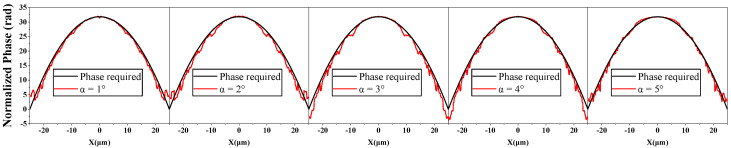
The required phase profile (black curve) and the computed phase profile of the metalenses (red curve) with different values of α.

For further analysis, we investigated the outgoing wavefront of the metalenses. As Figure 7 shows, when the value of α varies, the phase profile of the whole device vibrates around the required phase. It makes sense that the focal length changes little when α changes. As the value of α increases, the curve of the phase modulation seems to deviate from the normal one greater and greater (Figure 5a). Nevertheless, the proportions of the different diameter units in the lens are not the same, which is shown in Figure S6. We found that the metalens was mostly composed of units with a top diameter D greater than 200 nm. Therefore, we ignored the part where the top diameter D was smaller than 200 nm and adjusted the basepoint of the curves, as shown in Figure S7. As we can see, the deviation between the normal one and the deformation one first grows and then decays when α grows from 1° to 5°, whose trend is consistent with the wavefront’s and focus efficiencies’.

### 2.3. A Manufacturing-Tolerant Design (MTD) Method

According to the discussion above, we decided to ignore the insignificant influence of the rough surface in trenches and tried to find a method that can design based on the actual structure morphology to compensate for the phase deviation caused by the imperfect manufacturing process. This method consists of four steps and was named the manufacturing-tolerant design (MTD) method in this article. Before making a compensation, we need to estimate the phase modulation of the irregular units. Nevertheless, the fabricated structure is too complicated to model in the simulation, as shown in Figure 2. To solve this problem, we have found a convenient and feasible method to predict units’ modulation performance by just characterizing their cross-section.

**Figure 8:**
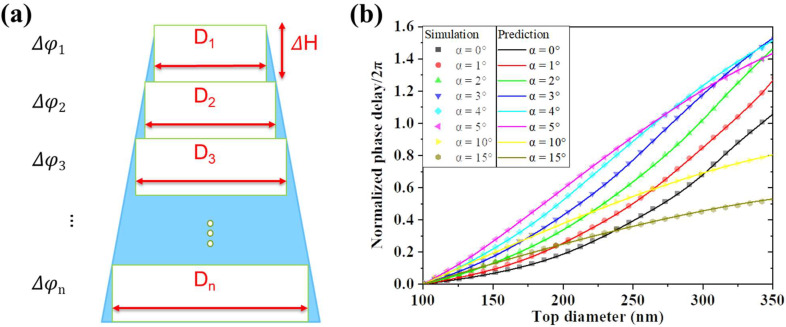
(**a**) The irregular-shaped grating unit is equivalent to a multi-piece structure with a graded gradient index; the truncated cone is divided into ultra-thin cylinders. (**b**) The phase modulation performance of the unit cells. The curves represent our prediction with the MTD method, while the symbols represent our previous simulation.

Firstly, we divided the unit structure into ultra-thin slices in the vertical direction. As for the unit model established in Section 2.2.2, each layer could be regarded as an ultra-thin cylinder with a diameter of D_i_ (i = 1, 2, 3, …, n) and a height of ∆H, as shown in Figure 8a, and when its diameter exceeds the period length p, it can be regarded as a cylinder with the diameter of Di truncated by a cube of side length p. Secondly, we built a phase-structure database of these regular cylinders and truncated cylinders with the height H the same way in Section 2.1 by the FDTD method. Thirdly, based on Equation (1), we can deduce the phase delay ∆φ of the divided unit cell by:

(3) $$\Delta \varphi =\frac{2\pi n_{eff}}{\lambda }\Delta H=\varphi \frac{\Delta H}{H}$$

The phase delay *φ* of the entire unit is obtained by accumulation:

(4) $$\varphi =\Delta \varphi_{1}+\Delta \varphi_{2}+\Delta \varphi_{3}+\ldots +\Delta \varphi_{n}$$

In this way, the performance of the irregular structures can be approached as long as the coordinates of the sidewall profile are characterized. Then, we made a simple verification with the FDTD method. We predicted the phase modulation performance of the unit cells in Section 2.2.2 as shown in Figure 8b. The curves represent our prediction, while the symbols represent our previous simulation results. It is apparent that they fit very well with a bit of deviation that may be caused by the calculation error. Finally, we used the predicted database to redesign the metalens in the same way mentioned in Section 2.1. Within these steps, metalenses consisting of the deformed unit structures mentioned in Section 2.2.2 were designed and the simulation results are shown below.

**Figure 9:**
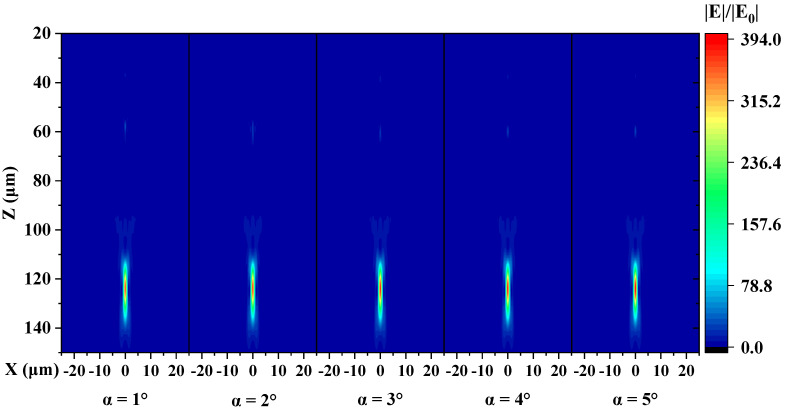
Simulated relative E-field intensity profiles in the xz-plane of the metalenses with different values of α designed by MTD method.

**Figure 10:**
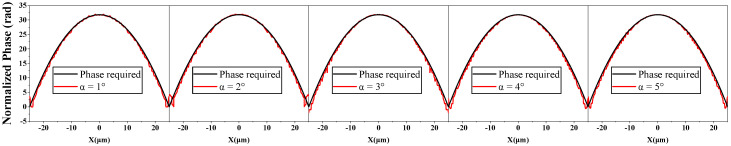
The required phase profile (black curve) and the computed phase profile of the metalenses (red curve) with different values of α after using the MTD method.

Figure 9 shows the intensity profiles simulation results of metalenses designed by the MTD method. As we can see, the relative E-field intensity distribution of the devices is close to the distribution of the ideal structure in Section 2.1. The amplitude of the E-field intensity at the focal spot of the metalens designed by the MTD method is much higher than before using it and even higher than that of the ideal structure. In addition, the discrepancy between the phase profile and the required one significantly declines after using the MTD method (Figure 10).

**Figure 11:**
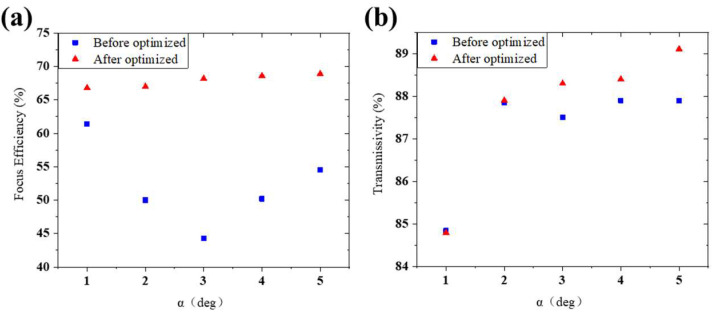
(**a**) Comparison of the focusing efficiency of the metalenses designed by MTD method (red triangles) and without using it mentioned in Section 2.2.2 (blue rectangles). (**b**) Transmissivity of metalenses.

As Figure 11a shows, the blue squares represent the focusing efficiency of the metalenses before compensation, and the red triangles represent the improved ones by the MTD method. Moreover, the improvement in efficiency is significant with using the MTD method. The results of the modified structures are all more than 66%. The larger value of α, the higher efficiency is achieved, which may be caused by the growth of transmissivity shown in Figure 11b. Figure S8 presents the normalized E-field intensity distribution of the focal spots of the metalenses mentioned above, while the dashed line indicates the diffraction limit. The distribution remains nearly the same as the diffraction limit when the structures vary in the condition we mentioned above. All these results prove that our MTD method greatly improves the performance of the devices with structural deformation. It is not reliable to measure our metalenses directly in the experiment, because their focal spots are in the substrate. When the light diverges from the focal spot and tries to come out of the substrate, it will be scattered by the interface and it is unpredictable in the experiment. Therefore, we are going to integrate metalenses with detectors to characterize their focusing efficiency in the next work. In addition, our work shows that metalenses with deformation structures have the potential to improve their performance significantly without improving the fabrication process. The actual fabricated structures will be much more complicated and irregular as Figure 2 shown. It is hard to build their actual models in the simulation and the functional relationship between the units and the phase shift can’t be calculated. Within this method, we can approach the phase control performance of the unit by calibrating the section of the fabricated unit structures. Then, metalenses with better performances can be designed.

## 3. Conclusions

In summary, we have investigated the impact on the metalens’ performance brought by the typical manufacturing imperfection through the FDTD method, which existed in our etching process development of InP material. We have found that the sidewall slope and ARDE effect greatly affected the focusing efficiency because of the phase modulation deviation, while the impact of the rough surface in trenches (rms < 30 nm) could be neglected. However, these phenomena change the focal length and the focal spot size little because the modulating phase of the whole device remains to vibrate around the required phase profile. In the end, we have presented a manufacturing-tolerant design (MTD) method to modify the phase profile deviation. Furthermore, it effectively improves the performance of the device with structural deformation, which was proved in simulation. It indicates that the device with the deformation structures may have the potential to improve its performance significantly. Although the actual fabricated structures will be much more complicated, with this method, we can approach the phase control performance of the unit by calibrating the section of the fabricated unit structures. Then, we can design metalenses with better performances without improving the fabrication process. The improvement would be more significant if the image recognition method were used. Our work is instructive for developing metalenses and can accelerate their integration application.

## Data Availability

The data presented in this study are available in article here.

## Supplementary Materials

The following supporting information can be downloaded at: https://www.mdpi.com/article/10.3390/mi13091531/s1, Figure S1: The schematic of the designed metalens’ top view, Figure S2: The rough surface in trenches (top view), Figure S3: The top view of the Figure 2c, Figure S4: The yellow region indicates that the base of the cones exceeds the unit period, Figure S5: SEM image of the etched pillars with deformation, some of the cones were merged with each other, Figure S6: Proportion histogram of the different diameter pillars in the metalens, Figure S7: The relationship between the phase and the top diameter D after adjusting the base point, Figure S8: The normalized electric field intensity distribution along the x-direction cutting through the focal spots on the focal plane (colorful lines) and the diffraction limit (dashed line).

## References

[1] Zhao R., Geng G., Wei Q., Liu Y., Zhou H., Zhang X., He C., Li X., Li X., Wang Y. (2022). Controllable Polarization and Diffraction Modulated Multi-Functionality Based on Metasurface. Adv. Opt. Mater..

[2] Ou K., Li G., Li T., Yang H., Yu F., Chen J., Zhao Z., Cao G., Chen X., Lu W. (2018). High efficiency focusing vortex generation and detection with polarization-insensitive dielectric metasurfaces. Nanoscale.

[3] Genevet P., Capasso F., Aieta F., Khorasaninejad M., Devlin R. (2017). Recent advances in planar optics: From plasmonic to dielectric metasurfaces. Optica.

[4] Sell J.Y.D., Doshay S., Yang R., Fan J.A. (2017). Large angle, multi-functional metagratings based on freeform multi-mode geometries. Nano Lett..

[5] Aieta F., Genevet P., Yu N., Kats M.A., Gaburro Z., Capasso F. (2012). Out-of-plane reflection and refraction of light by anisotropic optical antenna metasurfaces with phase discontinuities. Nano Lett..

[6] Li K., Haque S., Martins A., Fortunato E., Martins R., Mendes M.J., Schuster C.S. (2020). Light trapping in solar cells: Simple design rules to maximize absorption. Optica.

[7] Ren Z., Dong B., Qiao Q., Liu X., Liu J., Zhou G., Lee C. (2021). Subwavelength on-chip light focusing with bigradient all-dielectric metamaterials for dense photonic integration. InfoMat.

[8] Kun L., Tianyi G., Xiaoyong H., Qihuang G. (2021). On-Chip Nanophotonic Devices Based on Dielectric Metasurfaces. Chin. Opt. Lett..

[9] Hsiao H.-H., Chu C.H., Tsai D.P. (2017). Fundamentals and Applications of Metasurfaces. Small Methods.

[10] Ahmadivand A., Gerislioglu B. (2021). Photonic and Plasmonic Metasensors. Laser Photonics Rev..

[11] Zang W., Yuan Q., Chen R., Li L., Li T., Zou X., Zheng G., Chen Z., Wang S., Wang Z. (2020). Chromatic Dispersion Manipulation Based on Metalenses. Adv. Mater..

[12] Wang J., Zhang L., Zheng Y., Feng H.E., Qiu W. (2018). Design Method of Two-Dimensional Cylindrical Subwavelength Integrated Chip. J. Changchun Univ. Sci. Technol..

[13] Chen S.H., Yi X.J., Li Y., He M., Kong L.B., Ma H. (2001). Hybrid integration between long focus microlens array and IR detector array. Int. J. Infrared Millim. Waves.

[14] Abolmaali F., Brettin A., Green A., Limberopoulos N.I., Urbas A.M., Astratov V.N. (2017). Photonic jets for highly efficient mid-IR focal plane arrays with large angle-of-view. Opt. Express.

[15] Zhao Y.H., Chen M., Zhang R.Z. (2013). Influence of Chirp Parameter on Far-Field Distribution of the Beam. Opt. Optoelectron. Technol..

[16] Feng X., Deng J., Liu M., Li C., Zou D. (2017). Microlens array for shortwave infrared detectors. Opto-Electron. Eng..

[17] Feng Z.F.L., Hao B., Qing Y., Minjing L., Feng C. (2020). Development and Preparation of Refractive Infrared Microlens Array Device. Laser Optoelectron. Prog..

[18] Xu P., Qiao H., Wang R., Liu S., Li X. (2014). Study on Infrared Refractive Micro-lens Prepared by Dry and Wet Etching on CdZnTe Substrate. Semicond. Optoelectron..

[19] JLai J., Ke C.J., Chen S.H., Yi X.J. (2004). Fabrication of Microlens Array and Its Integration with Image Sensors. Semicond. Optoelectron..

[20] Chen S.H., Yi X.J., Kong L.B., He M., Wang H.C. (2002). Monolithic integration technique for microlens arrays with infrared focal plane arrays. Infrared Phys. Technol..

[21] Zhang S., Soibel A., Keo S.A., Wilson D., Rafol S.B., Ting D.Z., She A., Gunapala S.D., Capasso F. (2018). Solid-immersion metalenses for infrared focal plane arrays. Appl. Phys. Lett..

[22] Ou K., Yu F.L., Li G.H., Wang W.J., Miroshnichenko A.E., Huang L.J., Wang P., Li T.X., Li Z.F., Chen X.S. (2020). Mid-infrared polarization-controlled broadband achromatic metadevice. Sci. Adv..

[23] Chen W.T., Zhu A.Y., Capasso F. (2020). Flat optics with dispersion-engineered metasurfaces. Nat. Rev. Mater..

[24] Hou H., Zhang Y., Luo Z., Zhang P., Zhao Y. (2022). Design and fabrication of monolithically integrated metalens for higher effective fill factor in long-wave infrared detectors. Opt. Lasers Eng..

[25] Iliescu C., Chen B. (2008). Thick and low-stress PECVD amorphous silicon for MEMS applications. J. Micromech. Microeng..

[26] Pan Y.Q., Huang G.J. (2011). Study on Infrared Optical Properties of Amorphous Silicon Films Deposited by Ion Beam Assisted Deposition. J. Xi’an Technol. Univ..

[27] Volland B.E., Heerlein H., Kostic I., Rangelow I.W. (2001). The application of secondary effects in high aspect ratio dry etching for the fabrication of MEMS. Microelectron. Eng..

[28] Shi Z., Jefimovs K., Romano L., Stampanoni M. (2020). Towards the Fabrication of High-Aspect-Ratio Silicon Gratings by Deep Reactive Ion Etching. Micromachines.

[29] Gerlt M.S., Laubli N.F., Manser M., Nelson B.J., Dual J. (2021). Reduced Etch Lag and High Aspect Ratios by Deep Reactive Ion Etching (DRIE). Micromachines.

[30] de la Rue R.M., van der Heijden R.W., Viktorovitch P., Andriesse M.S.P., Carlstrom C.-F., Torres C.M.S., Midrio M., van der Drift E., Geluk E.-J., Karouta A.F. (2004). Deep dry etching process development for photonic crystals in InP-based planar waveguides. Photonic Cryst. Mater. Nanostructures.

[31] Wang H., Zhu Y., Gu Y., Chen P., Wang W., Chen X., Yang B., Li T., Shao X., Li X. (2020). High electron mobility InP grown by solid source molecular beam epitaxy. Mater. Sci. Semicond. Process..

[32] Dell’Olio F., Indiveri F., Innone F., Ciminelli C., Armenise M.N. Experimental countermeasures to reduce the backscattering noise in an InP hybrid optical gyroscope. Proceedings of the 2014 Third Mediterranean Photonics Conference.

[33] Khorasaninejad M., Zhu A.Y., Roques-Carmes C., Chen W.T., Oh J., Mishra I., Devlin R.C., Capasso F. (2016). Polarization-Insensitive Metalenses at Visible Wavelengths. Nano Lett..

[34] Dong X., Cheng J., Yuan Y., Xing Z., Fan F., Wang X., Chang S. (2022). Arbitrary large-gradient wavefront shaping: From local phase modulation to nonlocal diffraction engineering. Photonics Res..

[35] Fattal D., Li J., Peng Z., Fiorentino M., Beausoleil R.G. A silicon lens for integrated free-space optics. Proceedings of the Advanced Photonics.

[36] Arbabi A., Horie Y., Ball A.J., Bagheri M., Faraon A. (2015). Subwavelength-thick lenses with high numerical apertures and large efficiency based on high-contrast transmitarrays. Nat. Commun..

[37] Lalanne P., Chavel P. (2017). Metalenses at visible wavelengths: Past, present, perspectives. Laser Photonics Rev..

[38] Li F., Deng J., Zhou J., Chu Z., Yu Y., Dai X., Guo H., Chen L., Guo S., Lan M. (2020). HgCdTe mid-Infrared photo response enhanced by monolithically integrated meta-lenses. Sci. Rep..

[39] Yamamoto N. (2010). Selective Etching and Polymer Deposition on InP Surface in CH4/H2Reactive Ion Etching Using A New Mask Structure for Application to Submicrometer Pitch Grating. Appl. Phys. Express.

[40] Chen H.-Y., Ruda H.E., Navarro A.Z. (2001). Inductively coupled plasma etching of InP using N_2_/H_2_. J. Appl. Phys..

[41] Chen H.-Y., Ruda H.E. (2002). Inductively coupled plasma etching of InP using CH_4_/H_2_ and CH_4_/H_2_/N_2_. J. Vac. Sci. Technol. B Microelectron. Nanometer Struct..

