# Supplementary material for: The Impact of Manufacturing Imperfections on the Performance of Metalenses and a Manufacturing-Tolerant Design Method
Source: Micromachines (Basel). 2022 Sep 16;13(9):1531. doi: 10.3390/mi13091531 (PMC9501371; doi:10.3390/mi13091531)
Supplement: Supplementary file 1 [file micromachines-13-01531-s001.zip › micromachines-1866337-supplementary.pdf]

micromachines

MDPI

Supplementary Materials:

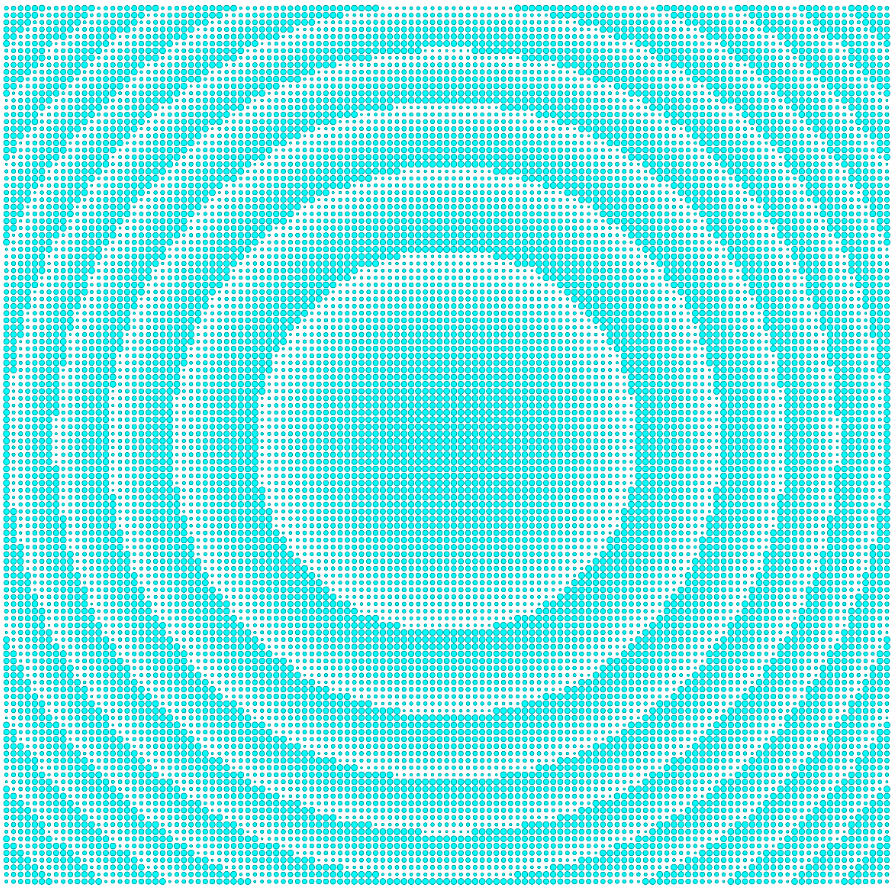

Figure S1. The schematic of the designed metalens' top view. Two different colors (cyan and white) are used to distinguish the pillars from the air.

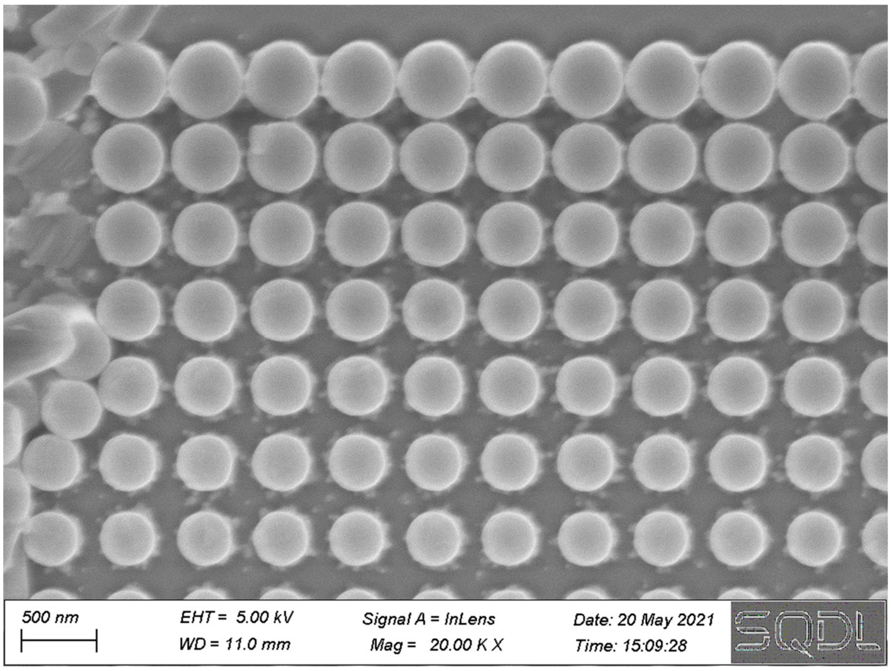

Figure S2. The rough surface in trenches (top view).

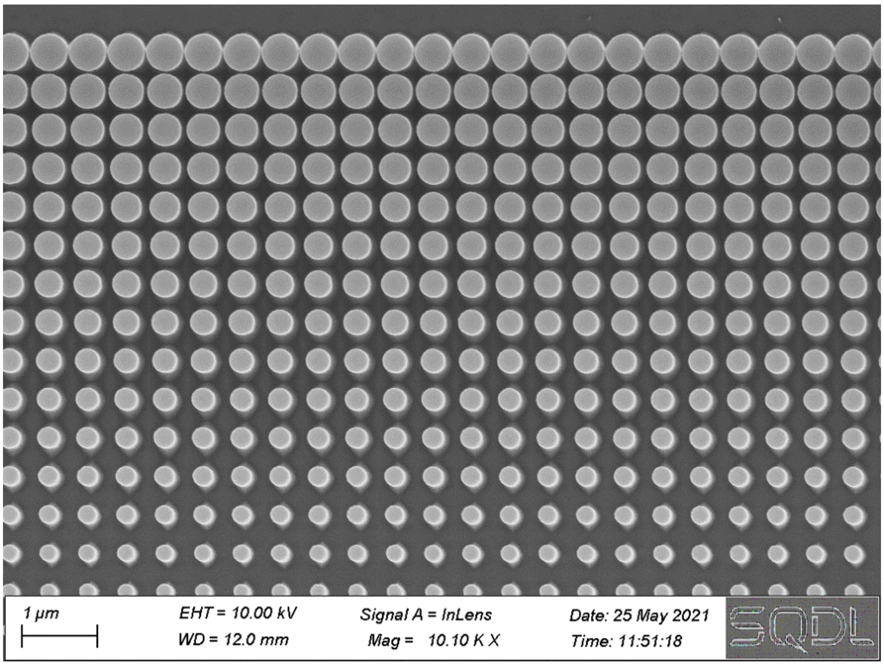

Figure S3. The top view of the Figure 2c.

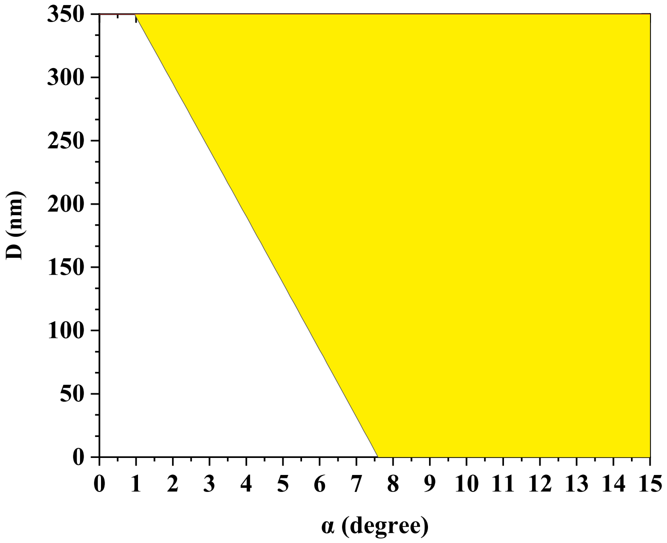

Figure S4. The yellow region indicates that the base of the cones exceeds the unit period.

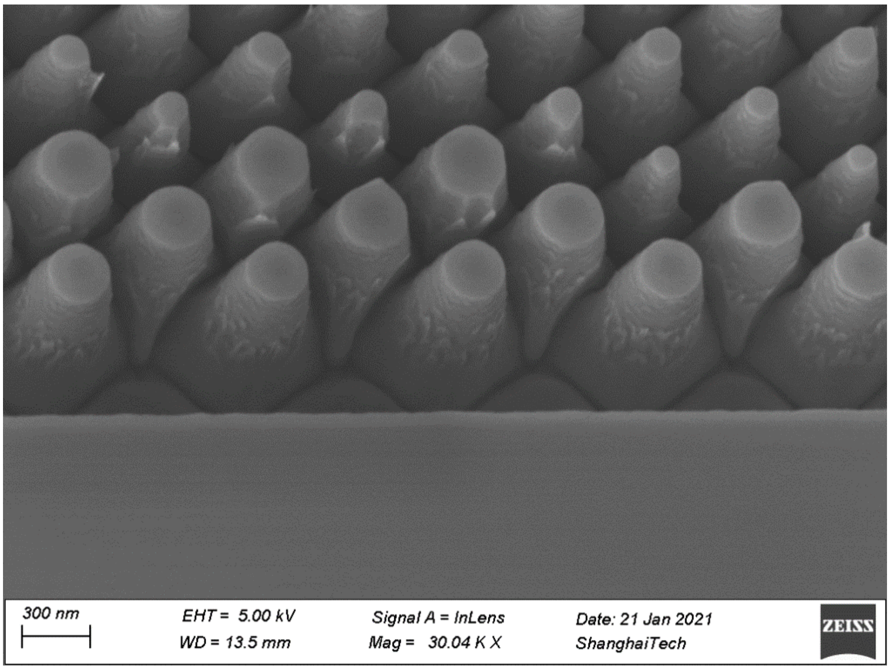

Figure S5. SEM image of the etched pillars with deformation, some of the cones were merged with each other.

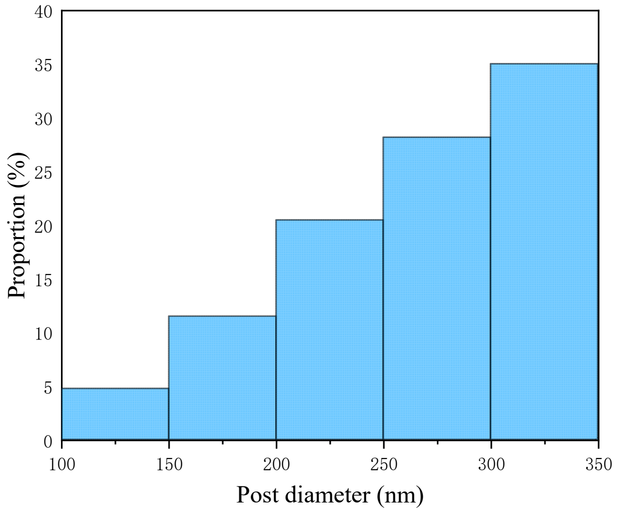

Figure S6. Proportion histogram of the different diameter pillars in the metalens.

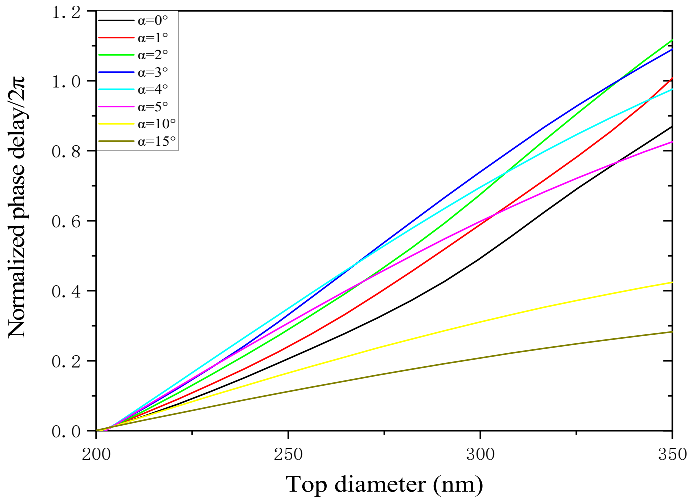

Figure S7. The relationship between the phase and the top diameter D after adjusting the base point.

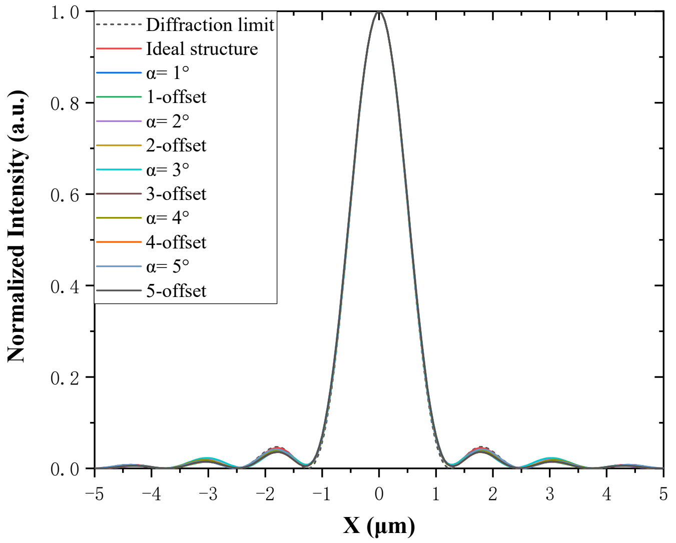

Figure S8. The normalized electric field intensity distribution along the x-direction cutting through the focal spots on the focal plane (colorful lines) and the diffraction limit (dashed line).
